# Functional capacity in community-dwelling older adults maintained by a higher friend network than family network: implications from a two-year longitudinal study

**DOI:** 10.1186/s13104-022-06216-8

**Published:** 2022-10-11

**Authors:** Mai Takase, Kyo Takahashi, Ryogo Ogino, Ryoichi Nitanai, Tomoki Tanaka, Shinya Saisho, Jun Goto, Katsuya Iijima

**Affiliations:** 1 Institute of Gerontology, The University of Tokyo, 7-3-1 Hongo, Bunkyo-ku, 113-8656 Tokyo, Japan; 2 Research Team for Social Participation and Community Health, Tokyo Metropolitan Institute of Gerontology, 3-9-7 Itabashi, Itabashi-ku, 173-0004 Tokyo, Japan; 3 Department of Public Health, School of Medicine, Dokkyo Medical University, 880 Kitakobayashi, Mibu- machi, Shimotsuga-gun, Tochigi, 321-0293 Tochigi, Japan; 4 Graduate School of Teacher Education, Saga University, 1- Honjo-machi, 840-8502 Saga, Japan; 5 Research Center for Advanced Science and Technology, the University of Tokyo, 4-6-1 Komaba, Meguro-ku, 153-8904 Tokyo, Japan; 6 Sociology Department, Faculty of Letters, The University of Tokyo, 7-3-1 Hongo, Bunkyo-ku, 113-8656 Tokyo, Japan; 7 Department of Architecture, Tokai University, City of Hiratsuka, 4-1-1 Kitakaname, 259-1292 Kanagawa, Japan; 8 Institute for Future Initiatives, The University of Tokyo, 7-3-1 Hongo, Bunkyo-ku, 113-8656 Tokyo, Japan

**Keywords:** Functional capacity, Family network, Friend network, Community-dwelling older adults

## Abstract

**Objective:**

Maintaining a level of functional capacity is essential for healthy aging. In this research, the association between the change in the level of functional capacity and social network typology was explored over a two-year period. Participants were recruited from a community-based cohort study within Kashiwa City, Japan, and data from the years 2016 and 2018 were used. Cognitive functions, functional capacity, and social network typology were assessed using the Mini-Mental State Examination, the Japan Science and Technology Agency Index of Competence, and the Lubben Social Network Scale, respectively. Binomial logistic regression analysis was then conducted to evaluate the association of individuals’ personal network and their functional capacity.

**Results:**

Results showed that, when compared to the group with both a high family and friend network, the group of community-dwelling older adults with both a low family and friend network (OR: 0.58, 95% CI: 0.34-1.00), and the group with a high family but low friend network demonstrated a lower functional capacity (OR:0.47, 95% CI: 0.26–0.85). Active social participation, facilitated by a friend network, could be a contributing factor to the maintenance of functional capacity.

## Introduction

The percentage of older adult populations is increasing globally. According to the World Population Aging 2019, there were 703million persons aged 65 years or over within the global population, and this number is expected to double to 1.5billion by 2050 [1]. Previous gerontological studies have revealed that living in a place where one is familiar in their geriatric years is essential for healthy aging. For example, being able to continue living in a place where social interactions have been developed was found to be related to an optimistic mental outlook [2]. In order for one to continue their independent living, maintaining their functional capacity [3] is fundamental. Functional capacity is a general term that refers to various physical and mental functions necessary for older people to lead their daily lives [4]. However, little is known about the factors that can lower the level of functional capacity, and further understanding is, therefore, required to support independent living.

Meanwhile, the state of one’s social network is associated with multiple health outcomes (e.g., [5]) and functional capacity could also be affected by their social network condition. For example, the report by Fiori et al., suggests that an absence of family in the context of friends is less detrimental than the absence of friends in the context of family, with regards to depression [6]. Furthermore, Park et al., report that physical and mental health risks among older adults in South Korea are associated with their social network typology, or more specifically, that having a friend leads to a better state of self-rated health and a lower probability of depression [7]. As introduced above, previous studies have focused on common geriatric symptoms. Although such findings can be applied to the context of care for older adults, the association of an individual’s social network and functional capacity should be explored to determine risk of a discontinuation of healthy aging before contracting serious symptoms. Therefore, in this study, we aimed to explore the association of the social network and functional capacity of older adults. Social network was studied in detail in two ways: family and friend networks.

## Materials and methods

### Study design

A community-based cohort study was conducted in Kashiwa City, Japan (hereafter, the Kashiwa study). The Kashiwa study first recruited 2,044 community-dwelling older adults using random selection from the basic resident register of Kashiwa City in 2012. Data from the year 2016 were used as the baseline data, and that from the year 2018 as data for the 2-year follow up. Cognitive functions were used as the exclusion criteria. Those who scored below 24 in the Mini-Mental State Examination were removed from the analysis. The number of participants was 1,329 in 2016, which is the baseline of this analysis, and 875 in 2018 during the two-year follow up. Some reasons for dropout were overlaps with other plans, contraction of medical symptoms that prevent attendance, confinement to the hospital, or death. After removing those with bad cognitive function, the final number of traceable participants was 638. The Japan Science and Technology Agency Index of Competence (JST-IC) was used to assess the functional capacity in older adults [4]. A score of 12 or more was defined as the individuals having high functional capacity, and those with a score of less than 12 were assumed as having low functional capacity. To define the network typology, the independent variable (type of personal network) was classified into four groups using the Lubben Social Network Scale (LSNS-6) [8], which assesses social networks from the perspectives of family and friends. The median score of the family and friends networks were 11 each. Therefore, those with score of 11 or more were defined as having high friend or family networks, and those with score of less than 11 were defined as having low friend or family network. Finally, four groups were created: (1) having both a high family and friend network; (2) having a high family network but low friend network; (3) having a low family network but high friend network; (4) having both a low family and friend network.

### Statistical analysis

Binomial logistic regression analysis was conducted to evaluate the association between individuals’ social network and their functional capacity; JST-IC was used as the dependent variable. The group with a high network of both family and friends was used as the reference group. Age, sex, cohabitation, mental state, comorbidity, and JST-IC at the baseline were adjusted as covariates. After conducting analysis using the whole data, the same analysis was conducted after stratifying participants by their age. Based on the age range of medical care system of older adults in Japan, the participants were classified into 65–75 years of age, and 75 years and older.

## Results

Participant characteristics are shown in Table1. The mean age was 75.7 ± 4.8 years old, and 53.3% of them were male. Those living with others made up 85.9% of the study sample and 73.5% were free of hypertension, diabetes, or stroke. A high percentage of participants (87.6%) had a good psychological wellbeing. Regarding the social network typology, 41.0% of the sample had both a high family network and friend network, 17.4% had a high family network and low friend network, 15.9% had a low family network and high friend network, and 25.7% had both a low family network and friend network. High functional capacity was observed in 56.4% of the participants at the baseline.

**Table 1:** Participant Characteristics (N = 638)

Variable		n (%)
Age	65–75 years old	302(47.3)
	75 years and older	336(52.7)
Sex		
	Male	340(53.3)
	Female	298(46.7)
Cohabitant		
	Living with others	548(85.9)
	Alone	90(14.1)
Comorbidity^*2^		
	None	469(73.5)
	One or more	169(26.5)
Psychological wellbeing^*3^		
	Good	559(87.6)
	Bad	79(12.4)
Social network type at baseline^*4^		
	High family & high friend	261(41.0)
	High family & low friend	111(17.4)
	Low family & high friend	101(15.9)
	Low family & low friend	164(25.7)
Functional capacity at baseline^*5^		
	High	355(56.4)
	Low	274(43.6)

^*1^hypertension, stroke, and diabetes were used for comorbidity

^*2^defined based on cutoff of WHO5

^*3^LSNS-6: low is ≤ 10, high is 11≤

^*4^JST-IC: low is ≤ 11, high is 12≤

The binomial logistic regression analysis (Table2) showed that, compared to the reference group, the group with low family and friend network (OR: 0.58, 95% CI: 0.34–1.00), and that with high family but low friend network were associated with lower functional capacity (OR: 0.47, 95% CI: 0.26–0.85) two years later. Furthermore, the group over the age of 75 had a lower score of functional capacity when considering those with both a low family and friend network (OR: 0.40, 95% CI: 0.19–0.86) and a high family but low friend network (OR: 0.38, 95%CI: 0.17–0.87). This trend was not observed in the group under the age of 75.

**Table 2:** Logistic Regression Analysis of Network Group and Functional Capacity

Network Group		Whole		Under 75		Over 75	
Family	Friend	OR	95% CI	OR	95% CI	OR	95% CI
High	High	Ref	Ref	Ref	Ref	Ref	Ref
High	Low	**0.47**	**0.26–0.85**	0.52	0.21–1.31	**0.38**	**0.17–0.87**
Low	High	0.89	0.48–1.66	0.97	0.37–2.60	0.83	0.36–1.89
Low	Low	**0.58**	**0.34-1.00**	0.92	0.40–2.11	**0.40**	**0.19–0.86**

*OR: odds ratio, CI: confidence interval

^†^adjusted for baseline functional capacity, age, sex, cohabitant, psychological wellbeing, and comorbidity

^‡^bold numbers indicate *p* < 0.05

## Discussion

The findings showed that those with low friend networks were likely to have a lower functional capacity two years later. When stratified by age, participants younger than 75 maintained their functional capacity, despite the level of friend network.

The overall results showed that when compared to having a low family network, a low friend network more significantly affected the functional capacity of older adults. Fiori et al. reported that older adults with a friend network participated in a higher number of social activities as compared to those with only a family network [6]. Active social participation correlates with better health and well-being [9, 10]. For example, the frequency of social outing behavior is associated with a better functional state [10], and a similar trend could, therefore, be observed with the functional capacity. On the contrary, similar to results from another study [11], having only a high family support network could indicate a lower functional capacity, in that older adults might be under hospitable care from family members and rely on them. Furthermore, the decrease in functional capacity was not observed in those with a low friend network under the age of 75. The percentage of younger older adults participating in social activities is higher compared to those above 75 [12]. This suggests that younger older adults might still have access to resources such as places and opportunities to interact with others, and the effect of the level of friendship network on functional capacity could be reduced, as compared to those over 75.

The results from this study suggested the importance of arranging a place where older adults, especially those over 75, can attend and socialize with non-family members. For example, it has been noted that a community place within walking distance from the older adults’ residence contributed to maintaining a social network [13]. Approximately 73% of the participants were over the age of 75 and accessibility of a social place could have contributed to maintenance of their social network. Neighborhood interventions considering the functional ability of older adults could result in the maintenance of friend networks.

## Conclusion

The results from this study implied that maintaining a friend network is essential for better functional capacity in older adults, especially in those above the age of 75. Future community interventions could focus on preparing a place for friendly interactions that is easily accessible by older adults above the age of 75.

## Limitations

The present study has some limitations. First, data from a cohort study were used for the analysis, and the dropout rate of participants over time should be considered. We analyzed the data of older adults who attended the third and fourth waves of the cohort study; however, we were unable to trace older adults who dropped out, which may have resulted in biased results. Follow-ups of older adults who dropped out are necessary to further examine the effect of lower friend networks. Second, this study was conducted in an urban area of Japan. Additional studies are required to further discuss the effect of family and friend networks on health-related outcomes of older adults residing in rural areas, since environmental factors such as availability of transportation resources could be limited.

## Data Availability

The datasets generated and/or analyzed during the current study are not publicly available but are available from the corresponding author on reasonable request.

## Abbreviations

JST-IC: The Japan Science and Technology Agency Index of Competence.
LSNS-6: Lubben Social Network Scale.
OR: odds ratio.
CI: Confidence Interval.
